# Epigallocatechin-3-gallate(EGCG) suppresses melanoma cell growth and metastasis by targeting TRAF6 activity

**DOI:** 10.18632/oncotarget.12836

**Published:** 2016-10-24

**Authors:** Jianglin Zhang, Zhou Lei, Zunnan Huang, Xu Zhang, Youyou Zhou, Zhongling Luo, Weiqi Zeng, Juan Su, Cong Peng, Xiang Chen

**Affiliations:** ^1^ The Department of Dermatology, Xiangya Hospital, Central South University, Changsha, Hunan, China; ^2^ Hunan Key Laboratory of Skin Cancer and Psoriasis, Xiangya Hospital, Central South University, Changsha, Hunan, China; ^3^ Key Laboratory for Medical Molecular Diagnostics of Guangdong Province, Dongguan Scientific Research Center, Guangdong Medical University, Dongguan, Guangdong, China

**Keywords:** EGCG, TRAF6, ubiquitination, melanoma

## Abstract

TRAF6 (TNF Receptor-Associated Factor 6) is an E3 ubiquitin ligase that contains a Ring domain, induces K63-linked polyubiquitination, and plays a critical role in signaling transduction. Our previous results demonstrated that TRAF6 is overexpressed in melanoma and that TRAF6 knockdown dramatically attenuates tumor cell growth and metastasis. In this study, we found that EGCG can directly bind to TRAF6, and a computational model of the interaction between EGCG and TRAF6 revealed that EGCG probably interacts with TRAF6 at the residues of Gln54, Gly55, Asp57 ILe72, Cys73 and Lys96. Among these amino acids, mutation of Gln54, Asp57, ILe72 in TRAF6 could destroy EGCG bound to TRAF6, furthermore, our results demonstrated that EGCG significantly attenuates interaction between TRAF6 and UBC13(E2) and suppresses TRAF6 E3 ubiquitin ligase activity *in vivo* and *in vitro*. Additionally, the phosphorylation of IκBα, p-TAK1 expression are decreased and the nuclear translocation of p65 and p50 is blocked by treatment with EGCG, leading to inactivation of the NF-κB pathway. Moreover, EGCG significantly inhibits cell growth as well as the migration and invasion of melanoma cells. Taken together, these findings show that EGCG is a novel E3 ubiquitin ligase inhibitor that could be used to target TRAF6 for chemotherapy or the prevention of melanoma.

## INTRODUCTION

Melanoma is among the most aggressive human cancers, and the incidence of this disease has increased in recent decades worldwide. According to reported statistics, approximately 140 thousand new cases of melanoma are diagnosed each year, of which approximately 50 thousand result in death [1, 2]. Chronic exposure to solar ultraviolet (UV) radiation is a high risk factor for melanoma and non-melanoma skin cancers [3, 4]. Exposure to sunlight induces gene mutation, consequently activating the oncogenic pathway [5]. Mutations of BRAF (40-50%) and NRAS (15-20%) are observed in cutaneous melanomas [6–8] and have been identified from benign melanocytic cell growth to the metastatic melanoma stage [9].

TRAF6 is a member of the tumor necrosis factor receptor-associated factor (TRAF) family, which plays critical roles in signaling transduction pathway, including NF-κB and MAPK pathway [10–12]. TRAF6 comprises a highly conserved N-terminal RING finger domain, several zinc fingers and a C-terminal TRAF domain [13–15]. The RING domain is well documented to possess ubiquitin (Ub) E3 ligase activity [15–17], and the TRAF domain serves as a protein-protein interaction domain [18]. TRAF6 is a critical signal transducer that initiates NF-κB pathway activation in response to pro-inflammatory cytokines through its E3 ubiquitin ligase activity, which synthesizes Lys63 (K63)-linked poly-ubiquitination chains; thus, this protein functions together with the E2 Ubc13/Uev1A complex to mediate TAK1 or IKK activation [19–21].

Evidence indicates that the E3 ubiquitin ligase activity of TRAF6 exerts important functions in tumorigenesis. *TRAF6* is among the oncogenes that are amplified in lung cancer, and the knockdown of TRAF6 expression significantly attenuates cell growth, tumor formation and Ras-mediated tumor formation. Interestingly, H-Ras and K-Ras 12V, but not K-Ras17N, initiate TRAF6 E3 ubiquitin ligase activity; this finding suggests that TRAF6 is a downstream effector of the Ras-induced pathway and links the RAS and NF-κB signaling pathways [22]. Our previous results demonstrated that TRAF6 is over-expressed in clinical melanoma tissues and melanoma cell lines, such as SK-MEL-5 and -28. The knockdown of TRAF6 expression dramatically attenuates the malignant phenotype, thereby decreasing cell growth, colony formation, and invasion and migration in a lung metastasis mouse model and in a xenograft model. Furthermore, TRAF6 directly interacts with BSG, which is important for the expression of MMPs during melanoma metastasis and induces the ubiquitination of BSG [23]. Mutation of the TRAF6 ubiquitination sites in BSG blocks its ability to induce MMP-9 expression and reduces melanoma cell invasion [23]. Thus, TRAF6 represents a potential therapeutic target for the treatment of melanoma.

Tea is one of the most widely consumed beverages in the world. Many studies have shown that the consumption of tea, particular green tea, has benefits for treating human diseases, including Parkinson's disease, Alzheimer's disease, stroke and obesity [24–30]. Catechins, a major class of flavonoids in green tea, include epicatechin (EC), epigallocatechin (EGC), epicatechin-3-gallate (ECG), and epigallocatechin-3-gallate (EGCG) [31–33]. EGCG is the most abundant of the catechins and accounts for 50 - 80% of the total amount of catechins in green tea. The anti-neoplastic nature of EGCG has been widely shown in cell culture, animal models and clinical studies [34–37], its effects on diseases such as lung cancer, colorectal cancer, prostate cancer, stomach cancer, and liver cancer are known, but fewer studies have investigated the effects of EGCG on melanoma cells. In this study, we found that TRAF6 is a novel target of EGCG. First, we used a structure-based virtual screening to identify TRAF6 as a potential target of EGCG. Then, a pull-down assay showed that EGCG directly binds to TRAF6. Further, based on a computational interaction model, we found that EGCG binds to TRAF6 at the residues of Gln54, Gly55, ILe72, Cys73, Asp57 and Lys96, and that this binding may destroy the association of TRAF6 with UBC13 (E2), thereby leading to the loss of its E3 ubiquitin ligase activity. Next, our results demonstrated that EGCG suppresses the E3 ubiquitin ligase activity of TRAF6 *in vitro* and *in vivo*. Consequently, the regulation of NF-κB pathway activation by TRAF6 was blocked after treatment with EGCG. Furthermore, the malignant phenotype of melanoma (including cell growth, invasion and migration) was dramatically attenuated by EGCG.

## RESULTS

### The binding mode between EGCG and TRAF6

**Figure 1:**
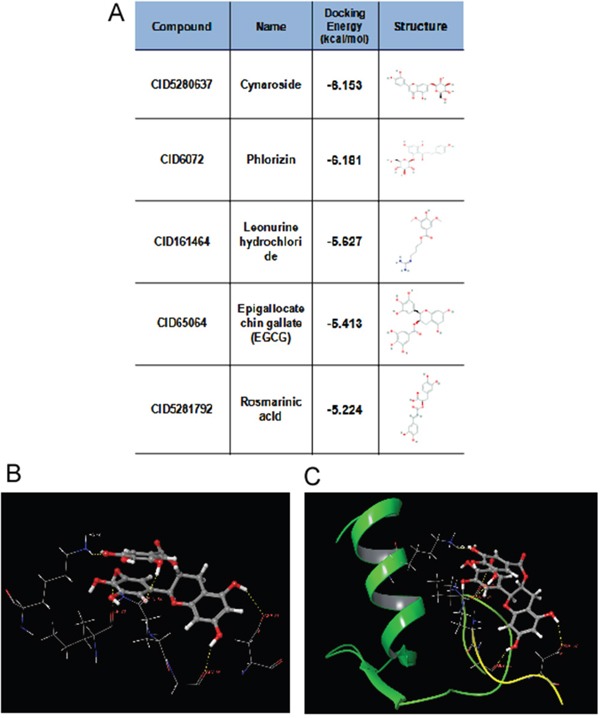
Predicted model of the EGCG-TRAF6 complex **A.** Listed the top five compounds obtained from the virtual screening of Shilan database to the structure of TRAF6 N-terminal domain. Column I gives the compound ID from PubMed compound database. Column II displays their commercial names. Column III lists the docking energy between each compound and the target protein. Column IV shows the structures of these ranked compounds, which were acquired from (http://www.ncbi.nlm.nih.gov/pubmed/). **B** and **C.** Hydrogen bonds between EGCG and five residues of Gln54, Gly55, Asp57, ILE72 and Lys96 at the interface of TRAF6 N-terminal domain with Ubc13. Note: the α-helices are drawn as cylinders and the β-strands as arrows. EGCG is shown in stick model and protein residues are shown in line model. The Figureures were generated by using Maestro [71].

Our study showed that TRAF6 plays a critical role in melanoma and is a potential chemotherapy or prevention molecular target [23]. Therefore, potential inhibitors of TRAF6 could be identified using computer screening. As an E3 ubiquitin ligase, TRAF6 forms a complex (as shown in PDB id 3HCT [38]) with UBC13 (E2 enzyme) to ubiquitinate its substrates. Based on this crystal structure, we performed computer screening and found EGCG as a potential inhibitor of TRAF6 (Figure 1A). The computational model of EGCG - TRAF6 complex was shown in Figure 1B and 1C. EGCG bound with TRAF6 at the interface of its N-terminal domain with UBC13 (Figure 1B). As shown in Figure 1B and 1C, EGCG formed five hydrogen-bonds with TRAF6: three involved in the side-chain atoms of three residues as Gln54, Asp57 and Lys96, while the other two engaged with the backbone atoms of two residues as Gly55 and ILE72 (Figure 1C). These residues, located either within or nearby the RING domain of TRAF6, played an important role on TRAF6's strong binding with Ubc13 [38]. Thus, the computational results indicate that EGCG could show UBC13-competitive inhibitory effects to TRAF6 protein.

### EGCG directly binds to TRAF6

**Figure 2:**
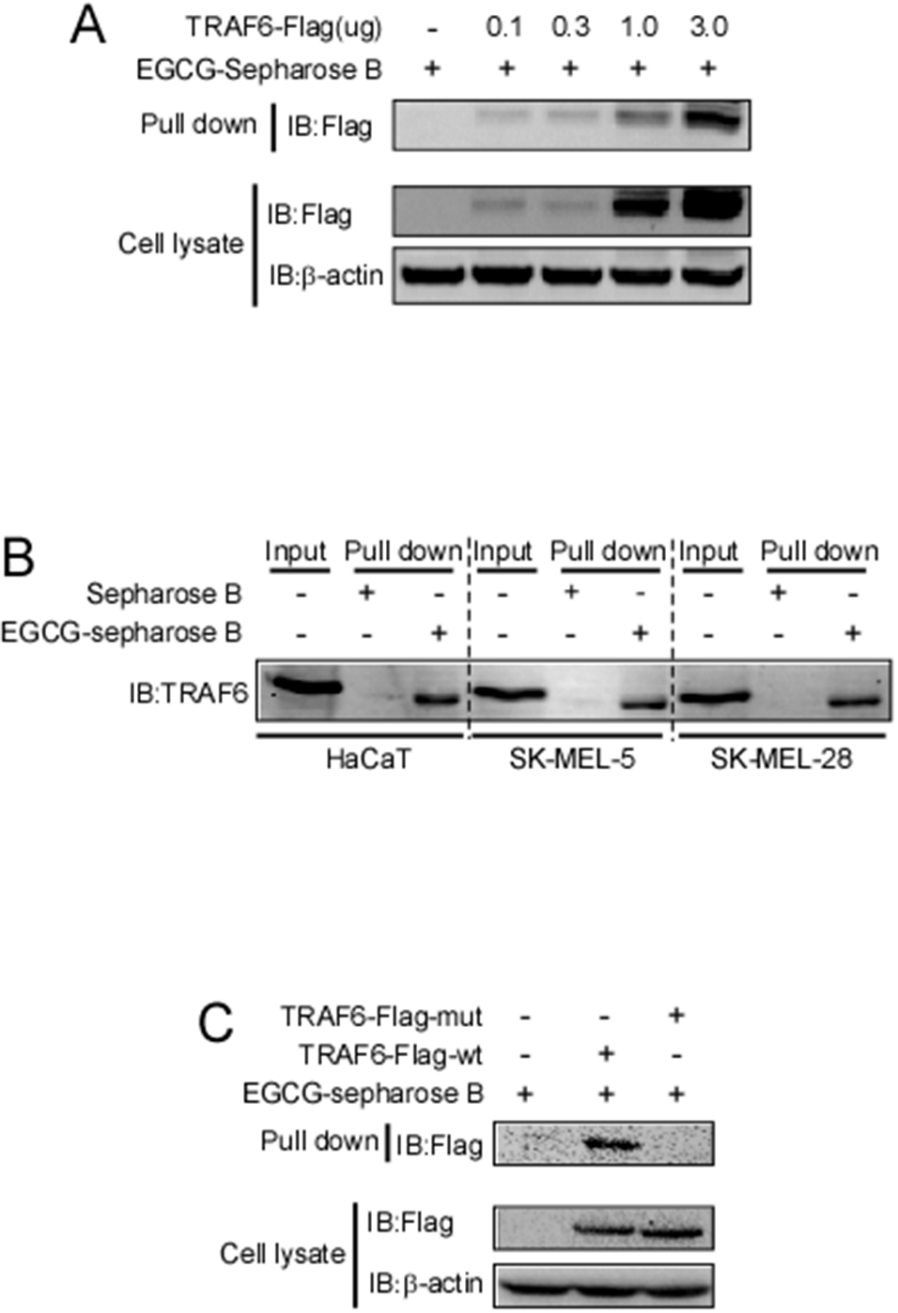
EGCG directly binds to TRAF6 **A.** Increasing amounts of TRAF6 plasmid were transfected into HEK293 cells. Thirty-six hours after transfection, the cell lysates were collected and incubated with EGCG-Sepharose 4B beads. This was followed by a pull-down assay as described in the *Materials and Methods* section. The precipitated complex was detected using an anti-Flag antibody. **B.** Lysates from HaCaT, SK-MEL-5 and SK-MEL-28 cells were incubated with Sepharose 4B-conjugated EGCG or Sepharose 4B-only beads, and a pull-down assay was performed following the protocol described in the *Materials and Methods* section. The complexes were subjected to immunoblotting to probe the interaction between EGCG and TRAF6. **C.** Wt and three sites mutated TRAF6 plasmid were transfected into HEK293 cells. Thirty-six hours after transfection, the cell lysates were collected and incubated with EGCG-Sepharose 4B beads. This was followed by a pull-down assay as described in the *Materials and Methods* section. The precipitated complex was detected using an anti-Flag antibody.

Next, we determined whether EGCG can directly bind to TRAF6. Increasing amounts of the TRAF6 plasmid were transfected into HEK293 cells, 36 h after transfection, cell lysates were collected and incubated with EGCG-Sepharose 4B beads followed by a pull-down assay. The result showed that EGCG dose-dependently bound to TRAF6 as shown in Figure 2A. To confirm that EGCG was associated with TRAF6, cell lysates collected from different cell lines (HaCaT, Sk-MEL-5, 28) were incubated with EGCG-Sepharose 4B beads. Western blotting revealed that TRAF6 bound to the EGCG-Sepharose 4B beads complex but not to the Sepharose 4B beads alone (Figure 2B). Based on our computer model (Figure 1B, 1C), EGCG might associate with TRAF6 at Gln54, Gly55, Asp57, ILE72, Cys73 and Lys96, therefore, to validate this interaction model, we mutated TRAF6 at Gln54, Asp57, ILe72 and mutation of TRAF6 lost the ability to associate with EGCG(Figure 2C), indicating these amino acids are key sites for EGCG bound to TRAF6.

### EGCG impairs the E3 ubiquitin ligase activity of TRAF6

**Figure 3:**
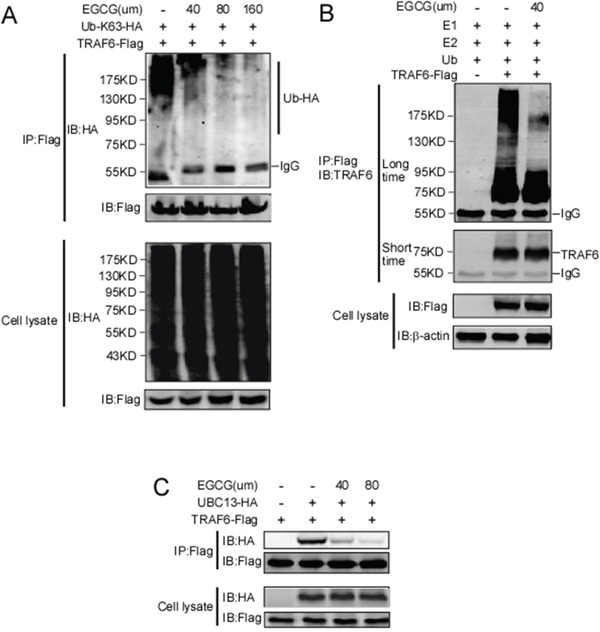
EGCG suppresses the E3 ubiquitin ligase activity of TRAF6 **A.** HEK293T cells were co-transfected with TRAF6-Flag and HA-K63-Ub. Forty-eight hours after transfection, the cells were treated with various doses of EGCG for 4 h as indicated. The cells were then harvested and lysates were immunoprecipitated using a Flag antibody. Auto-ubiquitinated TRAF6 was visualized using western blot analysis with an anti-HA antibody. **B.** Flag-TRAF6 was expressed in HEK293T cells, immunoprecipitated using an anti-Flag antibody, and preincubated with EGCG for 1 h. The beads were then incubated for 3 h at 37°C in reaction buffer (20 mM HEPES pH 7.4, 10 mM MgCl_2_, 1 mM DTT, 59 mM ubiquitin, 50 nM E1, 850 nM Ubc13, and 1 mM ATP). After incubation, the beads were centrifuged and washed four times with reaction buffer. The proteins were prepared in 6×SDS sample buffer and subjected to immunoblot analysis. **C.** Flag-TRAF6 and HA-UBC13 plasmids were transfected into HEK293 cells. After thirty hours transfection, the cells was treated with EGCG with different dosage for six hours as indicated, and then lysates were collected and immunoprecipitated with anti-Flag antibody, immunoblot was performed to test HA-UBC13 expression.

TRAF6 responds to K63-linked poly-ubiquitination, which is relevant to protein trafficking and signaling pathway activation [39], whereas K48-linked poly-ubiquitination leads to protein degradation [40]. Our computational model revealed that EGCG might associate with TRAF6 at the residues of Gln54, Gly55, Asp57, ILe72, Cys73, and Lys96 (Figure 1). Among these residues, Gln54, Asp57 and ILe72 have been reported to play a crucial role in the interaction of TRAF6 with UBC13 and that the mutation of these sites affects TRAF6 E3 ubiquitin ligase activity and NF-κB activation [38]. Given that EGCG is associated with TRAF6, we hypothesized that EGCG might affect its E3 ubiquitin ligase activity. TRAF6-Flag and HA-K63-Ub were co-transfected into HEK293 cells, 36 h after transfection, TRAF6 was immunoprecipitated with the Flag antibody, and TRAF6 auto-ubiquitination was detected using a HA antibody. TRAF6 E3 ligase activity is dependent on its auto-ubiquitination, particularly at K124. Mutations at K124 in TRAF6 impair the activity of TAK and IKK, subsequentlyabolishing NF-κB pathway activation [41]. Here, we found that TRAF6 auto-ubiquitination dose-dependently decreased with increasing EGCG (Figure 3A). To confirm the effect of EGCG on TRAF6 activity, an *in vitro* ubiquitination assay was performed. TRAF6 from the TRAF6-transfected HEK293T cell lysate was immunoprecipitated with the Flag antibody and was then incubated with E1, E2 or EGCG to study the reaction *in vitro*. E3 ligase activity was tested based on TRAF6 auto-ubiquitination. As shown in Figure 3B, auto-ubiquitination was dramatically blocked by EGCG, indicating that EGCG might suppress TRAF6 E3 ubiquitin ligase activity, moreover, we also proved that EGCG significantly attenuates TRAF6 interaction with UBC13 (Figure 3C).

### EGCG attenuates IL-1beta-induced NF-κB activation

**Figure 4:**
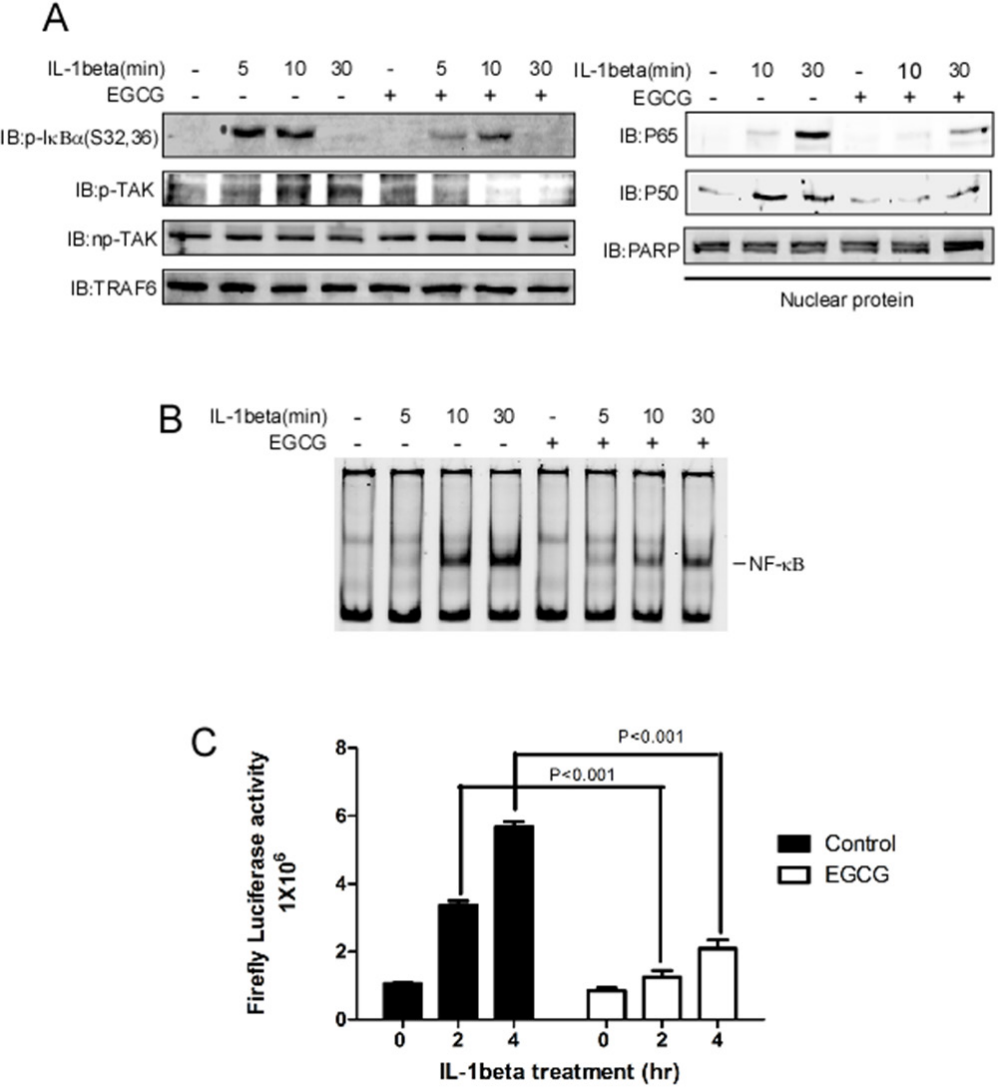
EGCG blocks IL-1beta-induced NF-κB pathway activation **A.** SK-MEL-5 cells were serum-starved for 16 h and treated with IL-1beta for 5, 10 or 30 min as indicated; whole cell lysates and nuclear fractions were then isolated. The phosphorylation levels of TAK1, p-IκBα TRAF6, and np-TAK1 were detected in the whole-cell lysate using specific antibodies, and β-actin was used as a loading control(Left panel). In the nuclear fraction, p65 and p50 were detected using specific antibodies. PARP was used as a nuclear protein marker and a loading control(Right panel). **B.** For the EMSA, the same nuclear extracts as those in (A) were incubated with a NF-κb DNA consensus sequence for 20 min. **C.** A *NF-κB-luciferase* reporter gene (0.4 μg) and a *Renilla* gene (0.2 μg) for normalization were co-transfected into SK-MEL-5 cells. At 20 h post-transfection, the cells were starved for 16 h and treated with IL-1beta for 2 or 4 h and EGCG as indicated. Firefly luciferase activity was determined in the cell lysates, and the values were normalized. Significant differences were evaluated using a one-way ANOVA, and the asterisk (*) indicates a significant difference (p < 0.05).

TRAF6 has been demonstrated to play a central role in IL-1beta-induced NF**-**κB activation. After treatment with IL-1beta, a ligand binds to IL-1beta receptor, and the adaptor protein MyD88 is recruited and forms a complex with the cytoplasmic domain of the receptor, thereby facilitating the activation of IRAK kinases [12]. Then, activated IRAK recruits TRAF6 to the ubiquitin-conjugating complex UBC13/UEV1A, which induces the K63-linked auto-poly-ubiquitination of itself or other proteins. The K63-linked chains that are ubiquitinated by TRAF6 act as a scaffold for TAB1 and TAB2, facilitating TAK1 activation [20]. Activated TAK1 phosphorylates and activates the IKK complex, thereby inducing the degradation of the NF-κB repressor IκBα and the nuclear translocation of p65 and p50. The data obtained in this study show that EGCG directly binds to TRAF6 and blocks its E3 ubiquitin ligase activity. Therefore, we investigated the effect of EGCG on the IL-1beta-induced activation of the NF-κB pathway. SK-MEL-5 cells were serum-starved for 16 h, pretreated with EGCG for 4 h, and then treated with IL-1beta. The cells were then harvested at various time points. Compared with results obtained using vehicle alone, the phosphorylation levels of IκBα and TAK1 were dramatically decreased in the presence of EGCG, however, total TAK1 and TRAF6 were unaltered (Figure 4A left panel). In the nuclear fraction, the IL-1beta-induced translocation of p65 and p50 was blocked after treatment with EGCG (Figure 4A, right panel). Next, an electrophoretic mobility shift assay (EMSA) and a luciferase reporter assay were performed to test the DNA binding and transcriptional activity of the NF-κB induced by EGCG. The results show that EGCG treatment significantly decreased the DNA binding activity (Figure 4B) and transcriptional activity (Figure 4C) of NF-κB, suggesting that EGCG attenuates the NF-κB activation induced by IL-1beta.

### EGCG blocks metastasis and growth of melanoma cells

**Figure 5:**
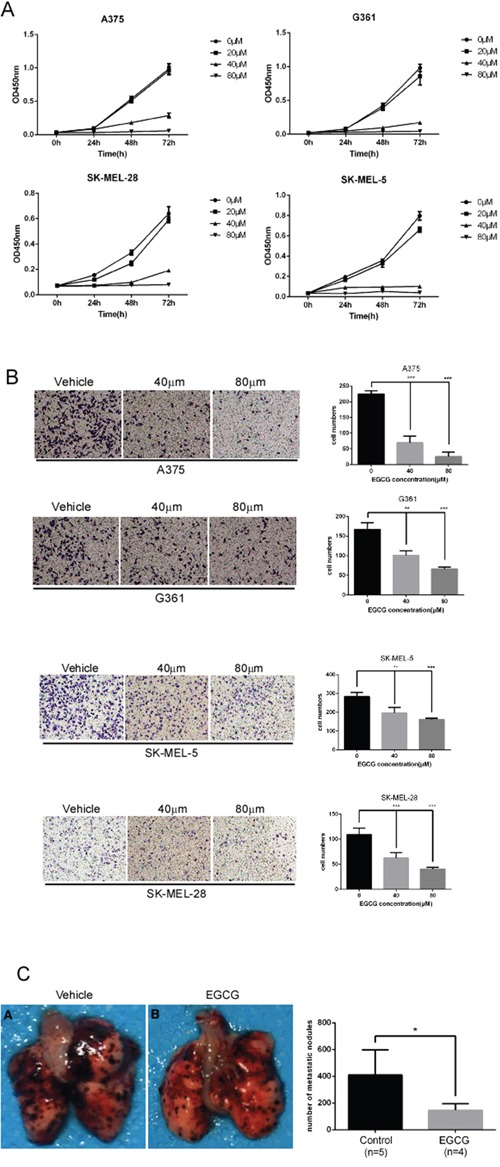
EGCG attenuates the migration, invasion and proliferation of melanoma cells **A.** SK-MEL-5, SK-MEL-28, A375 and G361 cells were seeded (1×103 per well/100 μL) into 96-well plates, and their proliferation was assessed using the CellTiter96 Aqueous One Solution Detection Kit. Data from multiple experiments are expressed as means ± S.D. Significant differences were evaluated using a one-way ANOVA, and the asterisk (*) indicates a significant difference (p < 0.05). **B.** Transwell experiments were performed as described in the *Materials and Methods* section. Invading cells crossing the membrane were stained with crystal violet(magnification, 100×). Each bar represents the means (n=3) ± SD of each group. **C.** lung metastasis mouse model was performed as described in the *Materials and Methods.* Average number of lung macro-metastasis per mouse from each group was determined. Representative macroscopic images of lung section were shown. Significant differences were evaluated using Student's t test, and the respective asterisks indicate significant differences (p < 0.05).

A previous study showed that TRAF6 knockdown inhibits the proliferation, invasion and migration of melanoma cells [23], indicating that TRAF6 plays a critical role in melanoma. The data obtained in this study revealed that EGCG can directly bind to TRAF6 and suppress its E3 ligase activity, thereby inactivating the NF-κB pathway. Based on these results, we hypothesized that EGCG might affect the malignant phenotype of melanoma. As expected, the number of migratory and invasive melanoma cells was significantly reduced after treatment with EGCG in different melanoma cell lines (Figure 5B), and the size and number of metastatic nodules in lung were dramatically reduced using the lung metastasis mouse model *in vivo* (Figure 5C). In addition, EGCG dramatically and dose-dependently inhibited cell growth in different melanoma cell lines (Figure 5A).

## DISCUSSION

Ubiquitination, one of the most important post-translational modifications, is the covalent attachment of ubiquitin to a lysine residue on a target protein to alter its biological function. This process occurs through the following three steps: the ATP-dependent attachment of ubiquitin (Ub) by a ubiquitin-activating enzyme (E1), the transfer of Ub from E1 to an active Cys site on a ubiquitin-conjugating enzyme (E2), and the transfer of Ub from E2 to the targeted protein or substrate using a Ub ligase (E3) [42]. According to recent studies, two E1 enzymes and approximately 50 ubiquitin-conjugating E2 enzymes exist, which control the type of ubiquitination that occurs. In the human genome, approximately 600 ubiquitin ligase E3 enzymes have been identified, and these enzymes decide substrate specificity [43]. In general, E3 ligases are divided into two major categories based upon their possession of a HECT (homologous to the E6-AP carboxyl terminus) domain or a RING domain. The HECT domain-containing E3s have an essential catalytic Cys residue and transfer Ub to the substrate through an intermediate that contains a thioester-linked Ub. In contrast, the Ring domain-containing E3s do not exert catalytic activity but interact with and bring E2s close to the substrate for ubiquitination [43, 44].

In the ubiquitination cascade, the E3 ligase is the final enzyme that determines the substrate specificity. Therefore, targeting E3 enzymes with a small-molecule inhibitor is expected to affect the pathways involving specific E3 ligases, thereby reducing toxic side effects. Currently, the search for specific E3 inhibitors (e.g., MDM2, IAP, and SKP2) is a promising strategy for chemotherapy or the prevention of cancer [45–48]. As mentioned above, TRAF6 has well-documented oncogenic characteristics. TRAF6 E3 ubiquitin ligase activity is required for the K63-linked ubiquitination of Akt and facilitates the membrane recruitment and consequent phosphorylation of Akt (T308) as well as its activation [49]. In TGFβ-induced prostate cell migration and invasion, TRAF6 E3 ubiquitin ligase activity has been shown to facilitate TbRI-ICD release and Lys63-linked polyubiquitination at K178 of TbRI, leading to its nuclear translocation and the subsequent regulation of associated genes [50, 51]. In our previous study, we demonstrated that TRAF6 is overexpressed in melanoma and regulates melanoma metastasis through the ubiquitination of CD147 to regulate the expression of MMP-9 [23]. Ectopic TRAF6 leads to myelodysplastic syndrome and facilitates fatal acute myeloid leukemia in primary bone marrow cells in mice [52]. In addition, TRAF6 is reported to increase HIF-1α expression and promote tumor angiogenesis [53].

TRAF6 is a RING domain-containing E3 ligase, and the first zinc finger and RING domain are responsible for the interaction between TRAF6 and UBC13(E2) [14, 41, 54]. In the RING domain, seven key residues, Glu69, Pro71, Ile72, Leu74, Met75, Ala101 and Pro106, were identified to form strong contacts with Ubc13. Gln54 and Asp57 in the first zinc finger also directly interact with UBC13. Furthermore, the mutation of Asp57 and Ile72 impairs the interaction between TRAF6 and UBC13 and inhibits the auto-ubiquitination of TRAF6 and its E3 ubiquitin ligase activity, subsequently blocking the IL-1beta-induced activation of the NF-κB pathway [38]. Compared with the RING domain-containing E3s, the HECT E3 ligases possess intrinsic catalytic activity and are more tractable drug targets than RING E3 ligases [55]. Thus far, most small E3 ligase inhibitors have been developed to target HECT-containing E3 ligases [56, 57]. In this study, we found that EGCG directly binds to TRAF6 (Figure 2), and our computational interaction model showed that EGCG probably associates with, Gln54, Ile72, Cys73, Gly55, Asp57 and Lys96 in TRAF6 (Figure 1), among these amino acids, mutation of Gln54, Asp57, ILe72 could impair associated with EGCG (Figure 2C). We also found that EGCG significantly blocks the E3 ubiquitin ligase activity of TRAF6 *in vivo* and *in vitro* (Figure 3A and 3B) and attenuates TRAF6 interaction with UBC13 (Figure 3C). Consequently, the phosphorylation of IκBα and TAK1 was decreased (Figure 4A). In turn, NF-κB pathway activation was dramatically attenuated (Figure 4B and 4C). Therefore, TRAF6 is a novel target molecule for EGCG, and our study provides another possible strategy to identify inhibitors for RING domain-containing E3 ligases by destroying the interaction between E3 and E2 enzymes.

EGCG is known to have anti-carcinogenic activity in various tumors, including lung cancer, liver cancer, and prostate cancer [58–60]. In this study, we found that EGCG significantly attenuated the proliferation and metastasis of melanoma (Figure 5), a finding that is consistent with the results of our previous study concerning the effects of TRAF6 in melanoma [23]. NF-κB is member of a family of proteins including RelA (p65), RelB and c-Rel [61] that form homo- or heterodimers and bind to specific sequences in promoter regions in response to extracellular stimuli, such as inflammation, cytokines, and tumor promoters. Aberrations of NF-κB play a critical role in tumorigenesis, including that of melanoma [62–66]. Several studies have shown that EGCG suppresses the NF-κB pathway by inhibiting the phosphorylation and subsequent degradation of IκBα, thereby preventing the nuclear translocation of p65 or p50 [67, 68]. By identifying molecules that are targeted by EGCG, a previous study indicated that the IKK complex is a direct target molecule of EGCG [69]. In this study, we provided a novel mechanism for EGCG in which this compound suppresses NF-κB pathway activation by directly binding to TRAF6 and inhibiting its E3 ligase activity, thereby blocking the nuclear translocation of p65 and p50.

Taken together, the results obtained in this study show that EGCG is a novel E3 ubiquitin ligase inhibitor that targets TRAF6. We also found that EGCG can directly bind to TRAF6 and destroy its interaction with E2, possibly functioning to suppress the E3 ligase activity of TRAF6. In turn, NF-κB pathway activation and the malignant melanoma phenotype are attenuated by EGCG. Given the safety of tea consumption, EGCG may be a novel pharmacological strategy for chemotherapy or the prevention of melanoma.

## MATERIALS AND METHODS

### Reagents and antibodies

Chemical reagents (including Tris, NaCl, and SDS) used in molecular biology and buffer preparation were purchased from Sigma-Aldrich (St. Louis, MO). Cell culture media and other supplements were purchased from Life Technologies, Inc. (Rockville, MD). EGCG was initially ordered from TianJin ShiLan technology company (China), but later it was purchased together with CNBr-Sepharose 4B and glutathione-Sepharose 4B beads from Sigma-Aldrich (St. Louis, MO). The TRAF6 antibody (Santa Cruz, CA, USA) was diluted to 1:500, the actin antibody (Santa Cruz, CA, USA) was diluted to 1:1,000, and the Flag antibody (Sigma, Germany) was diluted to 1:10,000. The HA antibody (Santa Cruz, CA, USA) was diluted to 1:500, (Cell Signaling Technology, Danvers, MA), and the TAK1 antibody (Cell Signaling Technology, Danvers, MA) was diluted to 1:1,000. Specific antibodies to detect p-TAK1, total TAK1, p65, p50, and phospho-IκBα (Ser32/Ser36) were obtained from Cell Signaling Technology, Inc. (Beverly, MA).

### Construction of expression vectors, cell culture and transfection

The expression constructs used in this study, including HA-Lys-63-ubiquitin and pCDNA3.0-Flag-TRAF6, have been described previously [23]. The human malignant melanoma cell lines SK-MEL-5, SK-MEL-28, A375, and G361 (American Type Culture Collection, Manassas VA, USA) and HEK293T cells (maintained in our laboratory) were grown in Dulbecco's modified Eagle's medium (DMEM, Thermo Scientific, MA, USA) supplemented with 10% fetal bovine serum (FBS) and antibiotics. All cell types were cultured at 37°C under a humidified atmosphere containing 5% CO_2_. For the transfection experiments, cells were transfected with various plasmids using the TurboFect Transfection Reagent (Thermo Scientific, MA, USA). The reagent and DNA were diluted in Opti-MEM (Invitrogen, CA, USA) and incubated for 15 min. The mixture was then added to cells growing in plates for 36 to 48 h to facilitate transfection.

### Immunoblotting and immunoprecipitation

Cells were lysed in RIPA buffer, and protein concentrations were determined using a BCA Protein Assay Kit (Santa Cruz, CA, USA). Proteins were immunoblotted using a standard protocol. For immunoprecipitation, extracts were precleared with 30 μL of agarose A/G beads (Beyotime Institute of Biotechnology) by rotary mixing for 1 h at 4°C. The beads were removed and another 30 μL of agarose A/G beads and 1.5 μg of the antibodies were added to the lysates. The mixture was then incubated on a rotary shaker overnight at 4°C. The beads were then washed twice in basic lysis buffer and then boiled for 10 min in loading buffer before loading on an appropriate SDS-PAGE. The blots were detected using an imaging system (Bio-Rad, USA).

### Virtual screening and molecular modeling

The crystal structure of TRAF6 N-terminal domain (PDB id 3HCT [38]) was downloaded from Protein Data Bank and further converted into an all-atom, fully prepared receptor model structure by using module Protein Preparation Wizard [70] in Maestro [71]. After that, the docking receptor grid was created by Glide's Receptor Grid Generation [72]. The grid box was set to have each side equaled 30 Å and the center of the box was determined by the residues surrounding the RING domain of TRAF6. We used a small compound database named as ShiLan database to perform compound screening. This database includes around 500 traditional Chinese medicine compounds, which are obtainable from TianJin ShiLan technology company (China). We downloaded their structures from PubMed compound database (http://www.ncbi.nlm.nih.gov/pubmed/) and built this database by using LigPrep module. Virtual screening of this compound database was carried out to the TRAF6 N-terminal domain by using Glide Docking [72, 73] in XP (Extra Precision) model [74]. For each compound, only the best scoring pose was retained in the output list after the docking. While Table 1 showed the best 5 compounds obtained from the screening, we selected the compound of EGCG (CID65064) for further experimental tests after their docking poses were manually viewed one by one.

**Table 1:** Showed the best 5 compounds obtained from the screening by ShiLan database including about 500 traditional Chinese medicine compounds

Compound	Name	Docking Energy (kcal/mol)	Structure
CID5280637	Cynaroside	−6.153	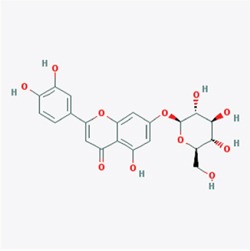
CID6072	Phlorizin	−6.181	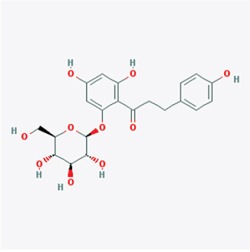
CID161464	Leonurine hydrochloride	−5.627	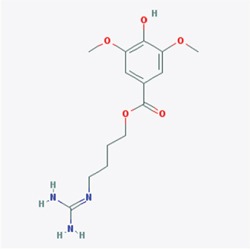
CID65064	Epigallocatechin gallate (EGCG)	−5.413	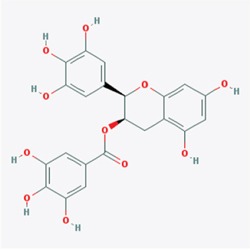
CID5281792	Rosmarinic acid	−5.224	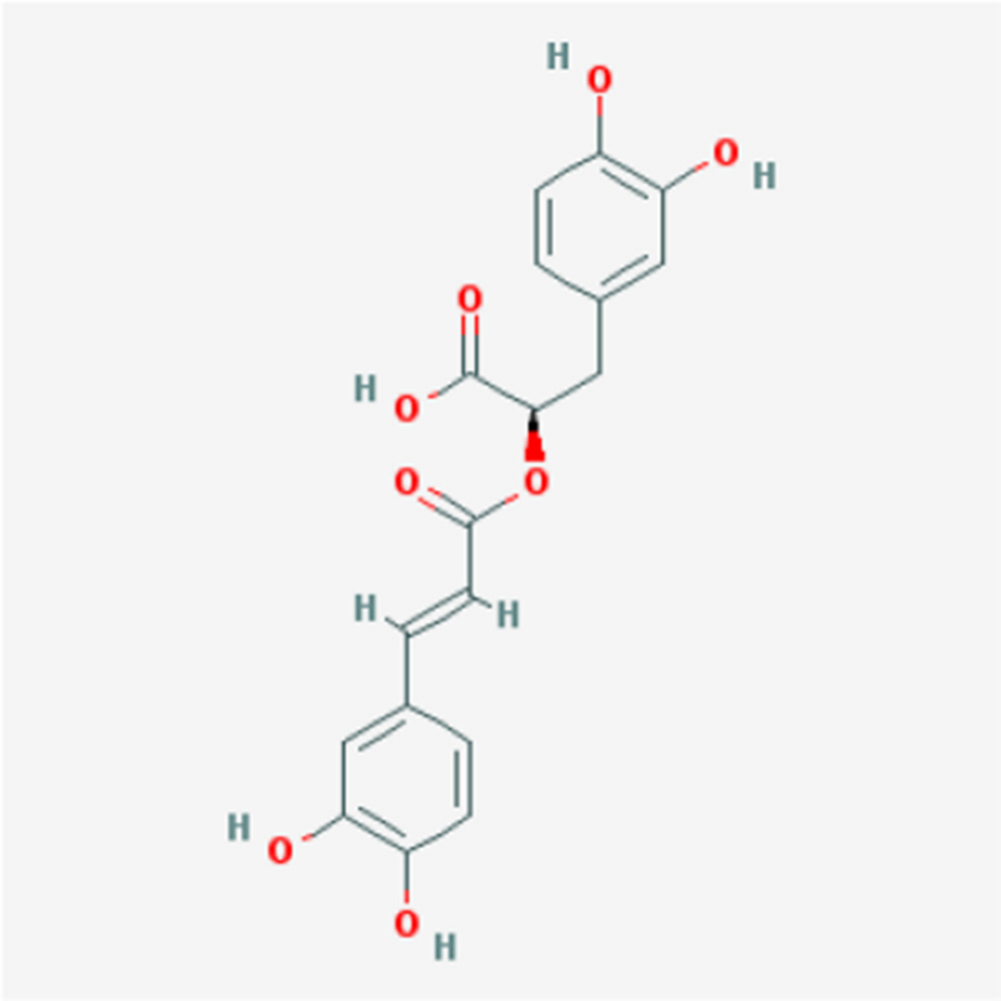

Table 1 listed the best 5 compounds obtained from the virtual screening of Shilan database to the receptor structure of TRAF6 N-terminal domain. Column I gives the compound ID from PubMed compound database. Column II displays their commercial names. Column III lists the docking energy between each compound and the target protein. Column IV shows the structures of these ranked compounds, which were acquired from http://www.ncbi.nlm.nih.gov/pubmed/. Hierarchical docking algorithm Glide [72] [75] [76] was also used for docking experiments to assess the possible binding mode between EGCG and the N-terminal domain of TRAF6. In order to enhance the conformational sampling, we used the ConfGen module [77] in the Schrödinger software package to generate 254 conformations of the EGCG for Protein-ligand docking. The binding pose with the lowest XP GScore (−7.952 kcal/mol) was considered as the correct binding structure and used as the representative docking structure between EGCG and TRAF6.

### *Ex vivo* pull-down assay

EGCG-linked Sepharose 4B beads were prepared according to the manufacturer's instructions (Amersham Biosciences) as described previously [78, 79]. The cell lysates (approximately 500 μg) were incubated with EGCG-linked Sepharose 4B beads (or Sepharose 4B alone as a control) in reaction buffer. After incubation with gentle rocking overnight at 4°C, the beads were washed three times with wash buffer, and the proteins that bound to the beads were analyzed by western blotting using Flag or TRAF6 antibodies.

### MTS assay

Cells (3.0×10^3^/well) were seeded in 96-well plates and then incubated for 24 h with various concentrations of EGCG for 24, 36 and 72 h. The effect of EGCG on cell viability was tested using a cell proliferation assay kit (Promega) according to the manufacturer's instructions. Assay solution was added to each well, and the absorbance (492 nm) was measured using a 96-well plate reader (Labsystem Multiskan MS, Labsystem, Finland). Each sample comprised 5 replicates.

### Preparation of cytosolic and nuclear fractions

SK-MEL-5 cells were seeded in 10-cm dishes and cultured to 90-95% confluence. The cells were serum-starved for 16 h, pretreated with EGCG for 4 h, stimulated with IL-1beta (20 ng/ml) and then harvested at various time points. The nuclear fraction was prepared using NE-PER nuclear extraction reagents following the manufacturer's suggested protocol (Pierce, Rockford, IL).

### *In vitro* ubiquitination assay

*In vitro* ubiquitination assays were performed as previously described [49, 80]. Briefly, Flag-TRAF6 was expressed in 293 T cells, immunoprecipitated using an anti-Flag antibody, and then incubated for 3 h at 37°C in reaction buffer (20 mM HEPES pH 7.4, 10 mM MgCl_2_, 1 mM DTT, 59 mM ubiquitin, 50 nM E1, 850 nM Ubc13 complex, 1 mM ATP, 30 mM creatine phosphate, and 1 U creatine kinase). After incubation, the beads were centrifuged and washed four times with reaction buffer. Proteins were eluted in 6×SDS sample buffer and subjected to immunoblot analysis.

### NF-κB gel shift assay

Nuclear fractions were extracted as described above. For the electrophoretic mobility shift assay (EMSA), 4 μg of nuclear protein was combined with 5x gel shift binding buffer (20% glycerol, 5 mM MgCl_2_, 2.5 mM EDTA, 250 mM NaCl, and 50 mM Tris-HCl), 0.25 mg/mL poly(dI)Σpoly(dC), and an IRDye 700-labeled NF-κB oligonucleotide (LI-COR, Lincoln, NE).

### Luciferase assay

Cells were transfected with *pNF-κB-Luc* and *SV-40-Renilla-Luc* (Promega, Madison, WI). At 20 h post transfection, the cells were serum-starved for 16 h, pretreated with EGCG for 1 h, and then treated with IL-1beta (20 ng/ml). The cells were then collected in passive lysis buffer. The cell lysates were analyzed for their firefly and *Renilla* luciferase activities using a dual luciferase assay kit (Promega).

### Transwell assays

To assay invasion, a Transwell experiment was performed by inserting an 8-μm pore chamber into 24 well plates (Corning, NY, USA). Matrigel (BD) was diluted (1:7) in serum-free DMEM and then added to each chamber and allowed to solidify completely. Transfected cell pellets were obtained and resuspended in serum-free medium at a density of 5 ×10^4^/100 μL and seeded in the upper chambers; 600 μL of DMEM containing 30% FBS was used as a chemotactic attractant at the bottom of the chamber. After incubation for 24 h, the cells were fixed with 4% paraformaldehyde in PBS and stained with crystal violet for 20 min. The Matrigel and cells at the top of the chambers were cleaned with a wet cotton swab. Invasive cells that remained at the bottom of the membrane were counted, and images were recorded using a microscope.

### Lung metastasis mouse model

The animal study protocol was approved by the Ethics Committee of Xiangya Hospital (Central South University, China). In the lung metastasis experiment, suspended B16F10 cells (0.5×10^6^ /0.1 mL) were injected into the lateral tail vein of 5-6-week-old male BALB/c (Shanghai SLAC Laboratory Animal Co. Ltd., Shanghai, China) mice. After injection one day, EGCG (100mg/kg) was injected into abdominal by three times a week. Animals were sacrificed after 10 days. Lung tissues were harvested and fixed in 10% buffered formalin.

### Statistical analysis

All quantified data are presented as means ± SEM of at least three experiments. Student's *t* test or one-way ANOVA was used to determine the significance of differences. A p value of less than 0.05 was considered statistically significant.
